# Synthesis of Borneol Mandelate and Its Acute Toxicity to Zebrafish

**DOI:** 10.3390/molecules31203595

**Published:** 2026-10-09

**Authors:** Rongxiu Qin, Weiqi Liu, Rusi Wen, Junkang Xie, Zhonglei Meng

**Affiliations:** 1 Guangxi Forestry Research Institute, Nanning 530002, China; qinrongxiu@gxlky.com.cn (R.Q.); liuweiqi@gxlky.com.cn (W.L.); wenrusi@gxlky.com.cn (R.W.); xiejunkang@gxlky.com.cn (J.X.); 2 Guangxi Laboratory of Forestry, Nanning 530002, China

**Keywords:** L-borneol, (R)-(-)-mandelic acid, titanium sulfate, borneol mandelate, acute toxicity testing

## Abstract

This study reports the synthesis of borneol mandelate from L-borneol, (R)-(-)-mandelic acid, and titanium sulfate, along with its characterization and acute toxicity assessment in zebrafish. The product was quantified by GC and structurally confirmed by IR, ^1^H-NMR, and ^13^C-NMR spectroscopy. Optimization of the reaction parameters revealed that the highest crude product purity (91.7% by GC) was achieved under the following conditions: chloroform as solvent, mandelic acid-to-borneol molar ratio of 1.5:1, titanium sulfate-to-borneol mass ratio of 0.15:1, and a reaction time of 9 h. Acute toxicity testing on zebrafish showed that borneol mandelate exhibited a maximum non-lethal concentration (MNLC) of 29.6 μM and a 10% lethal concentration (LC10) of 31.2 μM. Treated zebrafish displayed multiple adverse effects, including decreased blood flow, microphthalmia, hepatic atrophy, delayed yolk sac resorption, and behavioral disturbances.

## 1. Introduction

Borneol (C_10_H_18_O) is a bicyclic monoterpenoid alcohol crystallized from the resins or essential oils of Dipterocarpaceae plants [1,2], and exhibits a wide range of pharmacological activities [3,4]. L-Borneol, also known as Ai Pian, Ai Nao, or levo-borneol, is an ice-like crystalline solid and one of the two enantiomers of borneol. It is primarily obtained either by extraction from the natural plant *Blumea balsamifera* (Asteraceae) [5,6] or through chemical synthesis using camphor or turpentine as starting materials [7]. Natural D-borneol (dextroborneol), on the other hand, is a valuable medicinal and aromatic compound extracted from Lauraceae plants [8]. Borneol possesses multiple pharmacological properties, including antibacterial, anti-inflammatory, penetration-enhancing, cardiovascular, and cerebrovascular protective effects [9,10,11,12]. Owing to its natural origin, safety, and broad-spectrum antimicrobial efficacy, both L- and D-borneol hold great promise for diverse applications.

Borneol (Bing Pian) has been widely used in traditional medicine, yet its clinical application is constrained by its high volatility [13] and certain reproductive toxicity. To overcome these limitations, chemical modification of borneol has been pursued to develop derivatives with reduced toxicity and improved pharmacokinetic properties. Borneol esters, in particular, have drawn considerable attention due to their pharmaceutical significance. Notably, borneol-functionalized nanocarriers for targeted drug delivery have emerged as a promising research direction [14]. Bornyl acetate, an important ester derivative, serves not only as a fragrance ingredient and pharmaceutical intermediate but also as a major bioactive constituent of the volatile oil of *Amomum villosum*, with documented anti-inflammatory and immunomodulatory activities [15,16,17]. Esterification has been shown to attenuate the toxicity of borneol while enhancing its therapeutic effects, as demonstrated by a range of synthesized borneol esters, including danshensu acyl-alanyl-proline borneol ester, danshensu borneol ester, catechin borneol ester, gallic acid borneol ester, benzoic acid borneol ester, caffeic acid borneol ester, and ibuprofen borneol ester [18]. However, the synthesis of such ester derivatives is often hampered by severe steric hindrance, necessitating the use of condensing agents. For example, EDCI/DMAP has been employed for the synthesis of danshensu acyl-L-alanyl-L-proline borneol ester, whereas DCC has been used for gallic acid borneol ester [19,20]. Alternatively, 4-toluenesulfonic acid has been reported as an effective catalyst for the synthesis of 3,5-dinitrobenzoic acid borneol ester [21]. Additionally, titanium sulfate has been shown to catalyze the reaction between camphene and long-chain fatty acids to afford long-chain fatty acid isobornyl esters [22].

In recent years, the zebrafish (Danio rerio) has become a prominent vertebrate model for toxicological and pharmacological research. Over 70% of its genome shares orthologous sequences with that of humans, establishing it as a genetically manipulable system that mirrors significant drug-target interactions and pathways associated with human diseases [23,24]. In this study, the synthesis of borneol mandelate was carried out using titanium sulfate as a catalyst, and the product was obtained in high purity. The acute toxicity of the synthesized compound was subsequently evaluated in a zebrafish model. This study provides feasible molecular modification strategies and toxicological data support for the development of low-toxicity and high-safety functional borneol derivatives, and also offers important safety references for the subsequent pharmaceutical research, food-additive application, and environmental risk assessment of this compound.

## 2. Results and Discussion

### 2.1. Synthesis Reaction of Borneol Mandelate

#### 2.1.1. Synthesis Reactions Under Different Catalyst Conditions

At a molar ratio of borneol to mandelic acid of 1:1, a catalyst-to-borneol mass ratio of 0.15:1, a solvent volume of 60 mL toluene, and a reaction time of 9 h, the effects of different catalysts on the synthesis of borneol mandelate were investigated. The results are presented in Table 1.

As shown in Table 1, under different catalytic conditions, titanium sulfate exhibited a superior catalytic effect compared with boric acid, boron oxide, and sulfuric acid. The GC content of unreacted borneol in the product was relatively low, while that of borneol mandelate reached the highest value of 84.5%. Although the product catalyzed by sulfuric acid contained little borneol, it generated numerous by-products, resulting in a low yield. Moreover, the GC content of borneol mandelate was low, and the product appeared dark in color. The dark coloration increased the difficulty of decolorization and limited its potential applications. The acidity of titanium sulfate was greater than that of boric acid and boron oxide but lower than that of sulfuric acid. Therefore, a catalyst with moderate acidity was deemed optimal for this reaction. Titanium sulfate, a solid acid catalyst, was selected as it can be easily filtered and reused after the reaction.

#### 2.1.2. Synthesis Reactions Under Different Solvent Conditions

Using titanium sulfate as the catalyst, a molar ratio of borneol to mandelic acid of 1:1, a catalyst-to-borneol mass ratio of 0.15:1, and a reaction time of 9 h, the synthesis of borneol mandelate was conducted in various solvents. The results are summarized in Table 2.

As shown in Table 2, chloroform produced the best results, with no residual borneol detected and a borneol mandelate GC content of 86.7%. Toluene and cyclohexane, both less dense than water, allowed the timely removal of generated water during reflux. However, their boiling points—110 °C for toluene and 90 °C for cyclohexane—required higher energy input. In contrast, chloroform, being denser than water, required only 70 °C to reflux, substantially reducing energy consumption. Although the generated water could not be discharged immediately, it was temporarily stored in the water separator without affecting the forward shift of the reaction equilibrium.

#### 2.1.3. Synthesis Reactions Under Different Raw Material Ratios, Reaction Time, and Catalyst Dosage

The synthesis of borneol mandelate was further studied under varying conditions, including raw material ratios, reaction time, and catalyst dosage, using titanium sulfate as the catalyst and chloroform as the solvent. The results are shown in Figure 1.

As shown in Figure 1a, increasing the molar ratio of mandelic acid enhanced both the yield and the GC content of borneol mandelate. However, the improvement from a 1:1.5 ratio to 1:1.8 was marginal. Although an excess of mandelic acid promoted conversion and product yield, excessive quantities led to material waste and higher post-processing costs. Therefore, a molar ratio of 1:1.5 was adopted for subsequent experiments.

As presented in Figure 1b, increasing the catalyst dosage enhanced both product yield and GC content. However, when the catalyst-to-borneol mass ratio exceeded 0.20:1, further increases had little effect. Thus, 0.15:1 was determined as the optimal ratio.

As illustrated in Figure 1c, product yield increased with longer reaction time, while the GC content of borneol mandelate remained nearly unchanged. When comparing 9 h and 10 h reactions, the differences in both yield and purity were insignificant. Extended reaction times also resulted in more by-products, so 9 h was selected as the optimal duration.

The final optimized synthesis conditions for borneol mandelate were as follows: titanium sulfate as catalyst, chloroform as solvent, molar ratio of borneol to mandelic acid 1:1.5, catalyst-to-borneol mass ratio 0.15:1, and reaction time 9 h; the GC content of borneol mandelate reached 91.7%.

#### 2.1.4. Catalyst Reuse Test

Titanium sulfate was employed as the catalyst, chloroform as the solvent, with a molar ratio of borneol to mandelic acid of 1:1.5, a catalyst-to-borneol mass ratio of 0.15:1, and a reaction time of 9 h. After the reaction, the catalyst can be filtered and recovered for reuse. The results of two consecutive catalyst reuse cycles are presented in Table 3.

As shown in Table 3, after the catalyst was reused twice, the product yield decreased from 99.1% to 89.6%, while the GC content of borneol mandelate declined slightly from 91.7% to 90.8%. Although both yield and purity decreased, the change was minor, indicating that the titanium sulfate catalyst maintained high catalytic activity even after repeated use.

### 2.2. Isolation, Purification, and Structural Identification of Borneol Mandelate

#### 2.2.1. Separation and Purification of Borneol Mandelate

The borneol mandelate was purified by recrystallization to obtain colorless transparent crystals. GC analysis of the purified product showed its GC purity was above 99%, and the GC result is shown in Figure 2. The purified borneol mandelate was dissolved in absolute ethanol, and its specific rotation was determined to be −79.66° at 20 °C.

#### 2.2.2. Nuclear Magnetic Resonance Spectroscopy Analysis of Borneol Mandelate (^1^H NMR and ^13^C NMR)

The purified borneol mandelate sample (GC purity: 99%) was subjected to ^1^H NMR and ^13^C NMR analyses, and the results are shown in Figure 3.

^1^H NMR (600 MHz, DMSO) δ 7.45–7.40 (m, 2H), 7.35 (dd, *J* = 8.4, 6.7 Hz, 2H), 7.32–7.26 (m, 1H), 6.00 (s, 1H), 5.14 (s, 1H), 4.79 (ddd, *J* = 10.0, 3.5, 2.0 Hz, 1H), 2.29–2.20 (m, 1H), 1.73–1.61 (m, 3H), 1.18–1.07 (m, 2H), 0.92 (dd, *J* = 13.5, 3.5 Hz, 1H), 0.82 (s, 3H), 0.78 (s, 3H), 0.51 (s, 3H).

^13^C NMR (151 MHz, DMSO) δ 172.76, 139.99, 128.11, 127.80, 126.58, 79.10, 72.59, 48.58, 47.41, 44.21, 35.94, 27.54, 26.35, 19.41, 18.53, 13.04.

#### 2.2.3. Fourier-Transform Infrared (FT-IR) Spectroscopic Analysis of Borneol Mandelate

The purified borneol mandelate sample (GC purity: 99%) was subjected to infrared spectroscopic analysis, and the results are shown in Figure 4.

As shown in Figure 4, a strong absorption peak appeared near 3424 cm^−1^, attributed to the O-H stretching vibration mode, mainly from the hydroxyl group of mandelic acid. Absorption peaks in the 3100–3000 cm^−1^ region corresponded to the aromatic C-H stretching vibrations, while those between 3000 and 2800 cm^−1^ originated from the C-H stretching of saturated hydrocarbon and alkyl chains. The sharp band near 1732 cm^−1^ represented the C=O stretching vibration of the ester group, confirming that the hydroxyl group of borneol had successfully esterified with the carboxyl group of mandelic acid. The band near 1456 cm^−1^ corresponded to C-H bending vibrations in bridge-ring hydrocarbons. The absorption peak at about 1216 cm^−1^ was assigned to C-O stretching in the ester linkage, while the band at 1099 cm^−1^ reflected in-plane C-H bending in bridged and aromatic rings. The absorption near 752 cm^−1^ was associated with out-of-plane C-H bending vibrations in bridged and aromatic hydrocarbons. These characteristic peaks collectively confirmed the structural features of borneol mandelate.

### 2.3. Acute Toxicity of Borneol Mandelate to Zebrafish

#### 2.3.1. Determination of MNLC and LC_10_

The test results of maximum non-lethal concentration (MNLC) and 10% lethal concentration (LC_10_) for acute toxicity of borneol mandelate to zebrafish are shown in Table 4 and Figure 5.

As shown in Table 4 and Figure 5, the MNLC and LC_10_ of borneol mandelate for acute toxicity to zebrafish were 29.6 μM and 31.2 μM, respectively. Referring to the national standard” GB/T 21281-2007 Test method of fish acute toxicity for dangerous chemical products” [25], the obtained LC_10_ value is 31.2 μM (8.99 mg/L), which is close to the threshold of Acute Category 3 (10 < LC_50_ ≤ 100mg/L).

#### 2.3.2. Acute Toxicity Evaluation

The responses of zebrafish in each experimental group, including heart, circulatory system, hemorrhage and thrombosis, brain, eyes, liver, kidney, intestine, trunk/tail/notochord, muscle/somite and behavior, were observed under a dissecting microscope. Photographs of typical toxic-affected organs were collected, and their incidence rates (%) were statistically analyzed, as shown in Table 5 and Figure 6, Figure 7, Figure 8 and Figure 9.

As shown in Table 5 and Figure 6, Figure 7, Figure 8 and Figure 9, borneol mandelate exhibits certain acute developmental toxicity to zebrafish embryos. The earliest toxic manifestation of borneol mandelate in zebrafish embryos was yolk-sac absorption delay, which was detectable at 3.29 μM. At 9.87 μM, only exacerbation of yolk-sac absorption delay was noted, with no additional organ damage to the liver, circulatory system, or other tissues. Upon increasing the concentration to 29.6 μM, adverse phenotypes including reduced liver size, slowed blood flow, and abnormal behavior emerged, while no embryonic mortality occurred. At 31.2 μM, further malformations such as smaller eyes and trunk-tail curvature were observed, accompanied by 10% embryonic mortality. Notably, yolk-sac absorption delay reached 100% incidence, suggesting it is a relatively sensitive toxic endpoint. These results indicate that borneol mandelate can interfere with circulation, organ development, and substance metabolism of zebrafish embryos, thereby inhibiting normal embryonic growth and development.

## 3. Materials and Methods

### 3.1. Instruments and Reagents

The following analytical instruments were used in this study: the nuclear magnetic resonance spectrometer was an AVANCE NEO Ascend 600 MHz from Bruker (Billerica, MA, USA). A gas chromatograph (7890A series; Agilent Technologies, Santa Clara, CA, USA) equipped with quartz capillary chromatography columns (60 m × 0.25 mm × 0.25 µm) using AT-35 as the stationary phase was employed. Fourier Transform Infrared Spectrometer Nicolet iN10 (Thermo Fisher Scientific, Waltham, MA, USA) was used for IR analysis. The SGW-2 automatic polarimeter, with a test tube length of 100 mm and a light source wavelength of 589 nm, was obtained from Shanghai Precision Scientific Instrument Technology, Shanghai, China. An SZCL-2 digital intelligent temperature-controlled magnetic stirrer was purchased from Gongyi Yuhua Instrument Technology, Zhengzhou, China. The BJ210C electronic analytical balance was acquired from Prisys (Dietikon, Switzerland). LabRAM Soleil High-Resolution Microscopic Confocal Raman Spectrometer, HORIBA, Kyoto, Japan. SZX7 Stereomicroscope, OLYMPUS, Tokyo, Japan. VertA1 CCD Camera, Shanghai Tusen Vision Technology Co., Ltd., Shanghai, China. 6-Well Plate, Zhejiang Beilanbo Biotechnology Co., Ltd., Hangzhou, China.

The following materials and reagents were employed: L-borneol (98%), (R)-(-)-mandelic acid (98%), and titanium sulfate (96%) were purchased from Shanghai McLin Biochemical Technology (Shanghai, China). Chloroform, cyclohexane, and toluene were obtained from Sichuan Xilong Scientific Co., Ltd., Chengdu, China. Anhydrous sodium sulfate was supplied by Tianjin Damao Chemical Reagent Factory, Tianjin, China. Anhydrous sodium carbonate was purchased from Chengdu Kelong Chemical Co., Ltd., Chengdu, China. Dimethyl sulfoxide (DMSO) was obtained from Sinopharm Chemical Reagent Co., Ltd., Shanghai, China. Methylcellulose was supplied by Shanghai Aladdin Biochemical Technology Co., Ltd., Shanghai, China.

### 3.2. Experimental Methods

#### 3.2.1. Synthesis of Borneol Mandelate

L-borneol (15–20 g), (R)-(-)-Mandelic acid (15–30 g), catalyst (2.0–3.0 g), and solvent (60 mL) were added to a three-necked round-bottom flask. The mixture was magnetically stirred at 500 rpm under controlled temperature conditions, ensuring that solvent reflux carried away the water generated in the reaction. The reaction time was maintained between 6 h and 10 h. After completion, the catalyst was filtered out, and the filtrate was transferred to a separation funnel and washed up to four times with water. The lower organic layer was collected and subjected to rotary evaporation in a pear-shaped flask to remove the solvent. The residue was dissolved in ethyl acetate for GC analysis. Each experimental condition was repeated three times independently.

The reaction equations for the synthesis of different configurations of borneol mandelate are shown in Figure 10.

The product yield was calculated as:

$$\mathrm{yield}\;(\%)=\frac{m}{M}\times 100\%$$

where *m* represents the mass of crude borneol mandelate after drying (g), and *M* is the theoretical mass of borneol mandelate calculated from the initial loading amount of borneol (g).

#### 3.2.2. Purification of Borneol Mandelate

Borneol mandelate was purified via recrystallization. Petroleum ether with a mass ratio of 1:1 relative to the crude product was added to the synthetic product for recrystallization, and high-purity refined borneol mandelate was obtained after three repeated recrystallization cycles.

### 3.3. Analysis and Test Methods

#### 3.3.1. GC Analysis of Raw Materials and Products

Raw materials and products were quantitatively analyzed using gas chromatography, and component contents were determined by the area-normalization method. High-purity nitrogen served as the carrier gas. The temperature program was set as follows: initial temperature 70 °C (held for 2 min), ramped at 5 °C/ min to 150 °C (held for 3 min), followed by a ramp at 10 °C/min to 230 °C (held for 20 min). The inlet temperature was 250 °C. The total flow rate was 130.5 mL/min, with a split ratio of 50:1 and a septum purge rate of 3 mL/min. Detection was performed using a flame ionization detector with port temperature 250 °C, hydrogen flow rate 40 mL/min, air flow rate 450 mL/min, and nitrogen purge rate 25 mL/min. The injection volume was 0.5 µL.

#### 3.3.2. ^1^H NMR and ^13^C NMR Analysis of Product Spectra

The sample to be tested was dissolved in a deuterated solvent, transferred into an NMR tube, followed by selection of an appropriate frequency range, setting of experimental acquisition parameters, and acquisition of the spectrum.

^1^H NMR and ^13^C NMR spectra were recorded on a Bruker AVANCE NEO 600 MHz spectrometer. The sample was dissolved in DMSO-d_6_, and tetramethylsilane (TMS) was used as the internal reference for ^1^H chemical shift calibration. For ^13^C NMR, chemical shifts were referenced to the residual DMSO-d_6_ peak at δ 39.5 ppm. For ^1^H NMR: spectral width = 12 ppm, relaxation delay (D_1_) = 2.0 s, acquisition time = 2.5 s, number of scans (ns) = 16, pulse angle = 90°. For ^13^C NMR: spectral width = 220 ppm, relaxation delay (D_1_) = 2.0 s, acquisition time = 0.9 s, number of scans (ns) = 1024, pulse angle = 90°.

#### 3.3.3. Fourier Transform Infrared Spectroscopy Analysis

In a dry environment, a visible sample and an appropriate amount of dry potassium bromide powder were placed in a mortar and ground thoroughly several times. The mixture was then pressed into a transparent thin pellet using a tablet press. During testing, the background spectrum was first collected, followed by the infrared spectrum of the sample. The resolution was set to 4 cm^−1^ with 32 scans. The wavenumber range was 400/600–4000 cm^−1^.

#### 3.3.4. Determination of Specific Rotation

A 0.50 g sample was accurately weighed into a 5 mL volumetric flask, dissolved in anhydrous ethanol, and diluted to volume. The specific optical rotation was measured using sodium D-line light (589.3 nm) at 20 °C with a tube length of 1 dm.

#### 3.3.5. Acute Toxicity Test of Zebrafish

Zebrafish used in the experiment were maintained in culture water at 28 °C. The water was prepared by adding 200 mg instant sea salt to 1 L of reverse osmosis water, with the following water quality parameters: conductivity of 450–550 μS/cm, pH value of 6.5–8.5, and hardness equivalent to 50–100 mg/L CaCO_3_. Wild-type AB strain zebrafish at 2 days post-fertilization (2 dpf) were randomly distributed into 6-well plates, with 30 larvae per well for each experimental group. The test sample was dissolved in water to prepare stock solutions for aqueous exposure. A blank control group and a solvent control group were set up simultaneously, and each well contained 3 mL of test solution. The plates were incubated at 28 °C for 3 days. The number of dead zebrafish in each group was counted daily, and dead individuals were removed promptly. At the end of the exposure experiment, zebrafish in each experimental group were observed under a dissecting microscope for toxic responses including heart, circulatory system, hemorrhage and thrombosis, brain, eyes, liver, kidney, intestine, trunk-tail-notochord, muscle-somite, and behavioral performance. Representative photographs of toxic phenotypes were captured, and the incidence rate (%) of each phenotype was calculated. Origin 8.0 software was used to plot the optimal concentration-mortality response curve, from which the maximum non-lethal concentration (MNLC) and 10% lethal concentration (LC_10_) of the test sample toward zebrafish were computed.

## 4. Conclusions

The optimal conditions for the synthesis of L-borneol mandelate from (R)-(-)-mandelic acid were as follows: titanium sulfate as the catalyst, chloroform as the solvent, molar ratio of borneol to (R)-(-)-mandelic acid of 1:1.5, mass ratio of catalyst to borneol of 0.15:1, and reaction time of 9 h. Under these conditions, the GC content of the obtained borneol mandelate reached 91.7%. Catalyst recycling experiments were performed under the optimal conditions. After two cycles of reuse, the product yield decreased from 99.1% to 89.6%, while the GC content of borneol mandelate only dropped from 91.7% to 90.8%, demonstrating that the catalyst still maintained relatively high catalytic activity after two reuses.

L-borneol mandelate synthesized from (R)-(-)-mandelic acid was purified by recrystallization, and the GC content of the purified product was above 99%. The purified product was dissolved in absolute ethanol, and its specific rotation was determined to be −79.66° at 20 °C.

The MNLC and LC_10_ of borneol mandelate for zebrafish acute toxicity were 29.6 μM and 31.2 μM, respectively. Referring to the national standard “GB/T 21281-2007 Test method of fish acute toxicity for dangerous chemical products”, the obtained LC_10_ value is 31.2 μM (8.99 mg/L), which is close to the threshold of Acute Category 3 (10 < LC_50_ ≤ 100 mg/L). Toxic phenotypes including decreased blood flow, smaller eye size, reduced liver volume, delayed yolk-sac absorption, and abnormal behavior were observed in exposed zebrafish embryos.

## Figures and Tables

**Figure 1:**
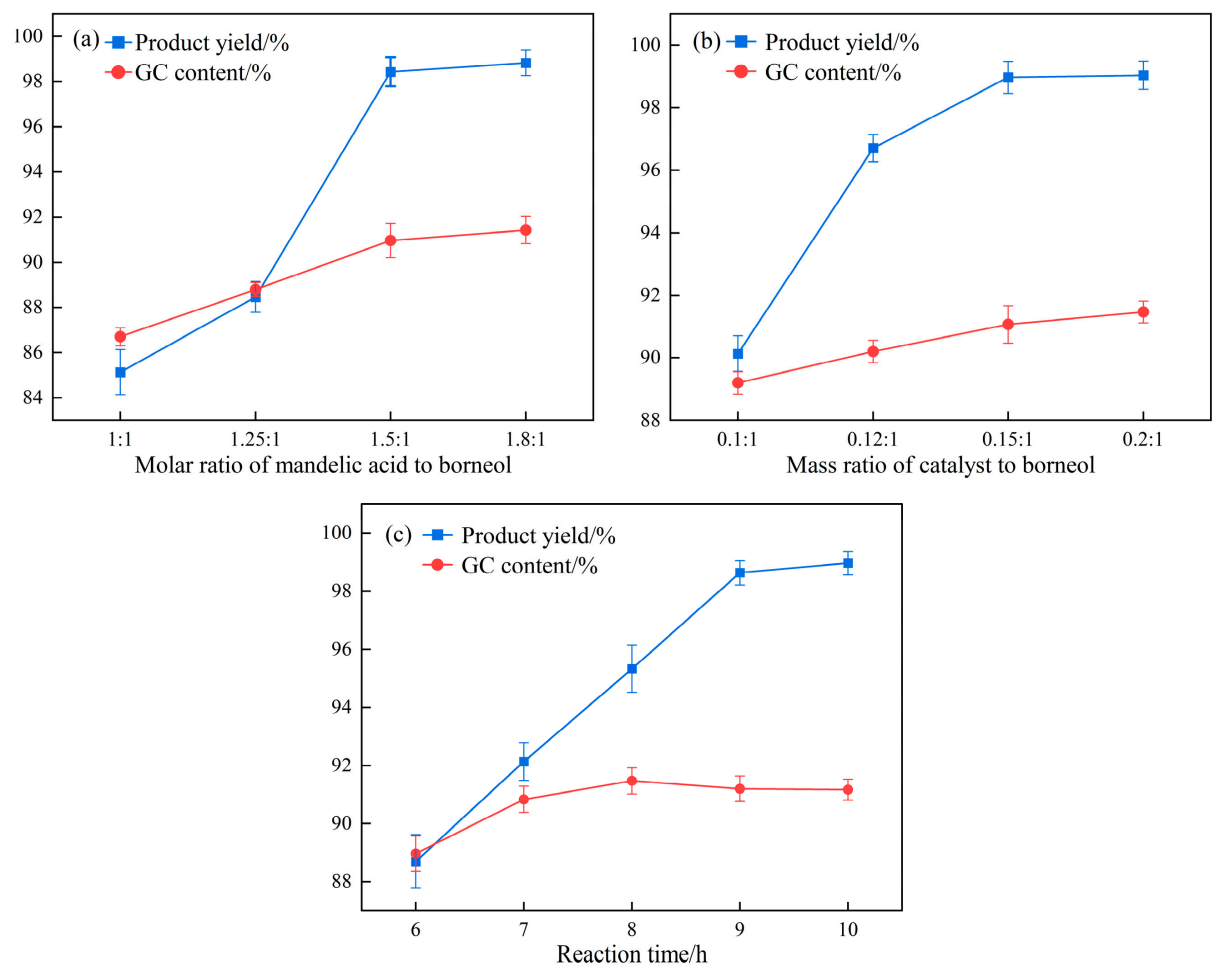
Synthesis reactions under different reaction conditions. Note: (**a**) shows the product yield and GC content of borneol mandelate under different raw material ratios; (**b**) shows the product yield and GC content of borneol mandelate under different catalyst dosages; (**c**) shows the product yield and GC content of borneol mandelate under different reaction times.

**Figure 2:**
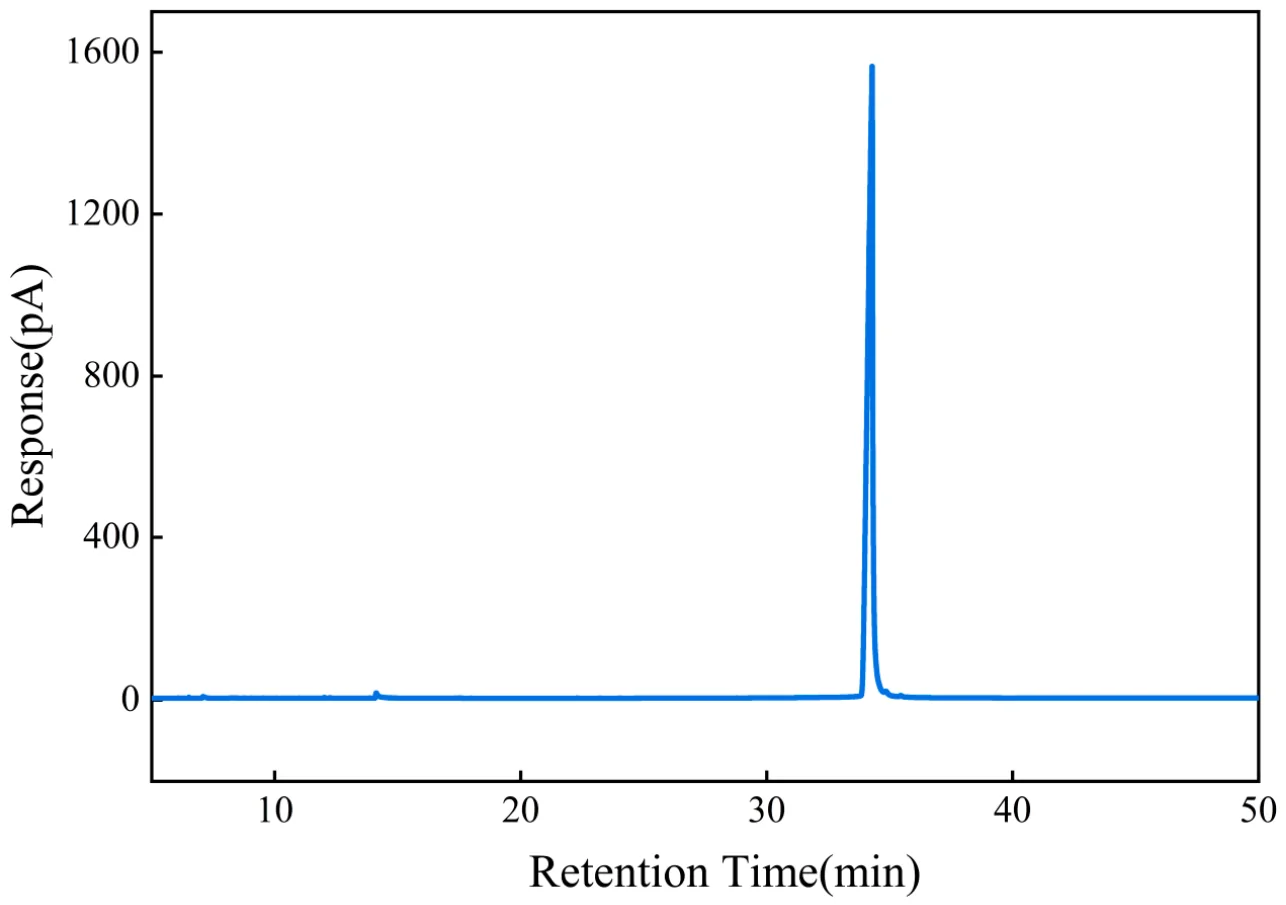
GC chromatogram of borneol mandelate.

**Figure 3:**
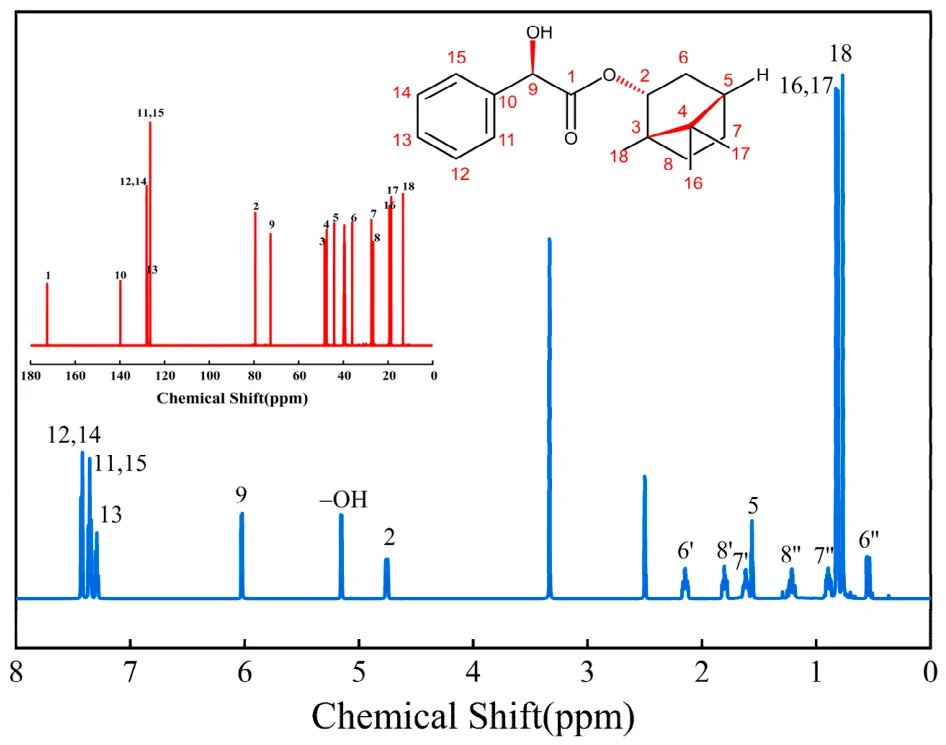
NMR spectra of borneol mandelate.

**Figure 4:**
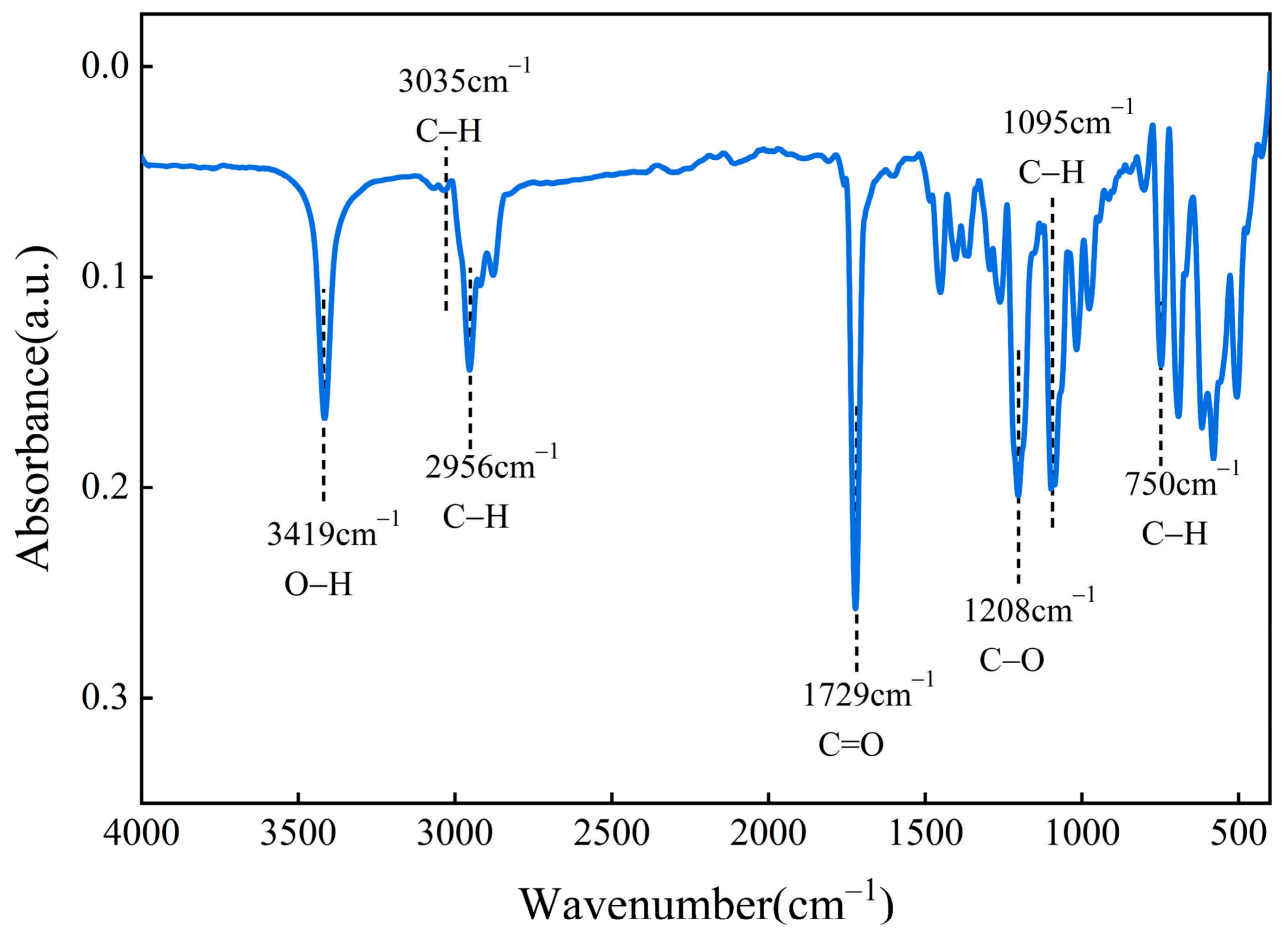
FT-IR characteristic spectrum of borneol mandelate.

**Figure 5:**
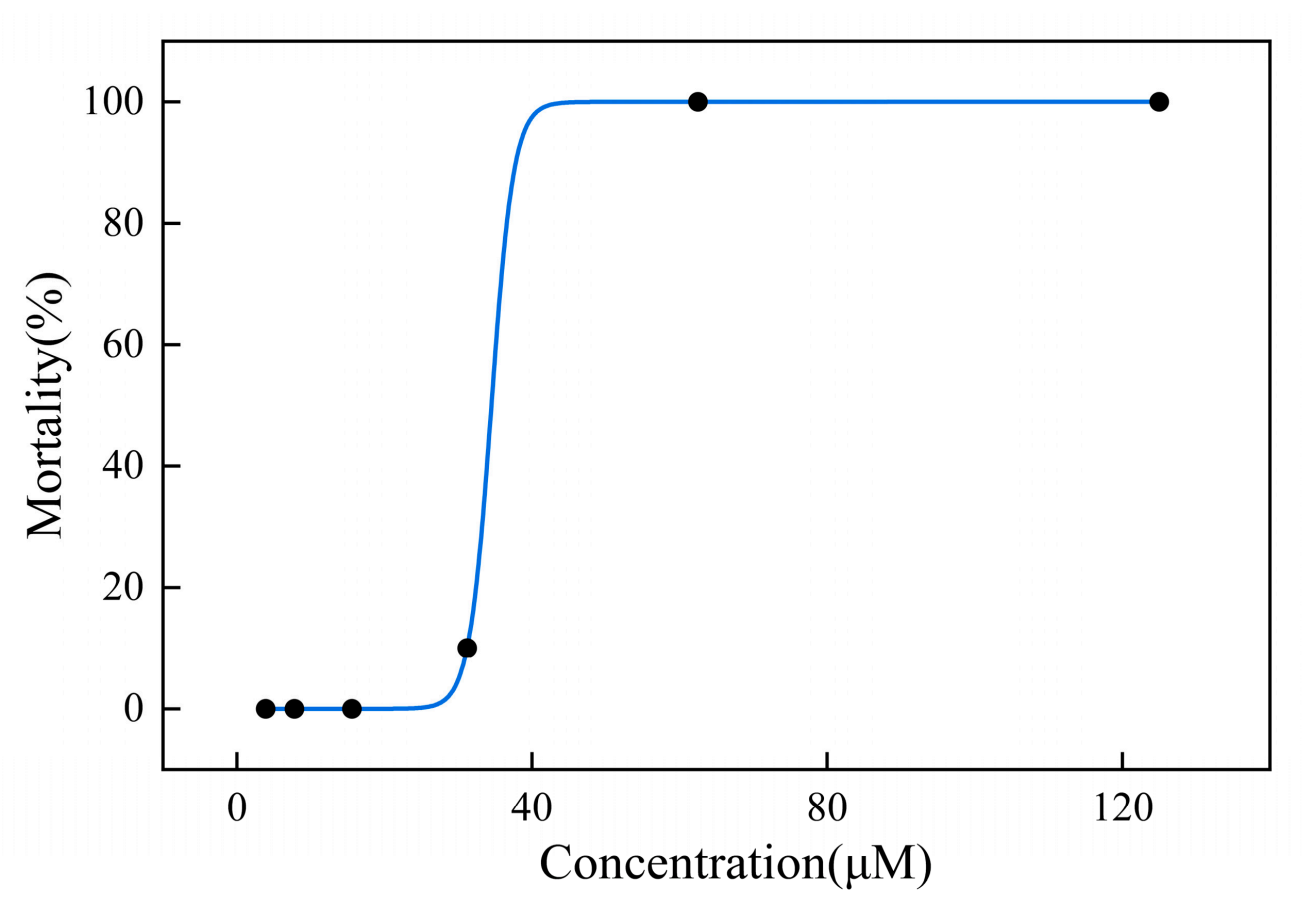
Concentration-mortality curve after sample treatment.

**Figure 6:**
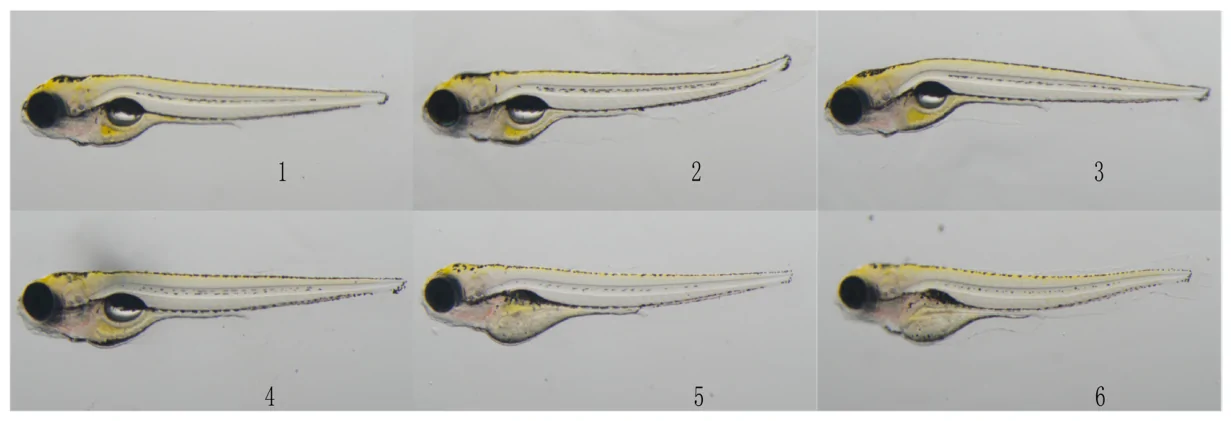
Phenotypes of zebrafish embryos after sample treatment-Whole-body view. Note: 1—Normal control group; 2—Solvent control group; 3—borneol mandelate 3.29 μM; 4—borneol mandelate 9.87 μM; 5—borneol mandelate 29.6 μM; 6—borneol mandelate 31.2 μM.

**Figure 7:**
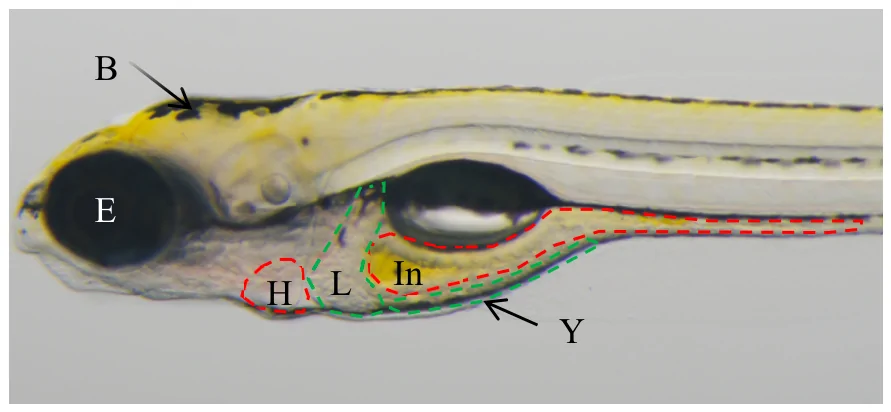

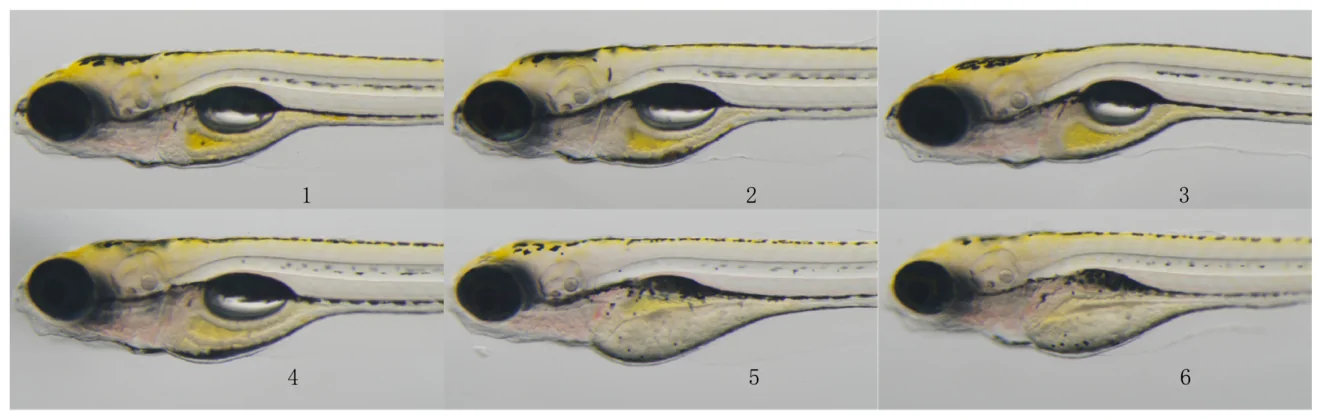
Phenotypes of zebrafish embryos after sample treatment-Half-body view. Note: B = brain; H = heart; In = intestine; L = liver; E = eye; Y = yolk sac; 1—Normal control group; 2—Solvent control group; 3—borneol mandelate 3.29 μM; 4—borneol mandelate 9.87 μM; 5—borneol mandelate 29.6 μM; 6—borneol mandelate 31.2 μM.

**Figure 8:**
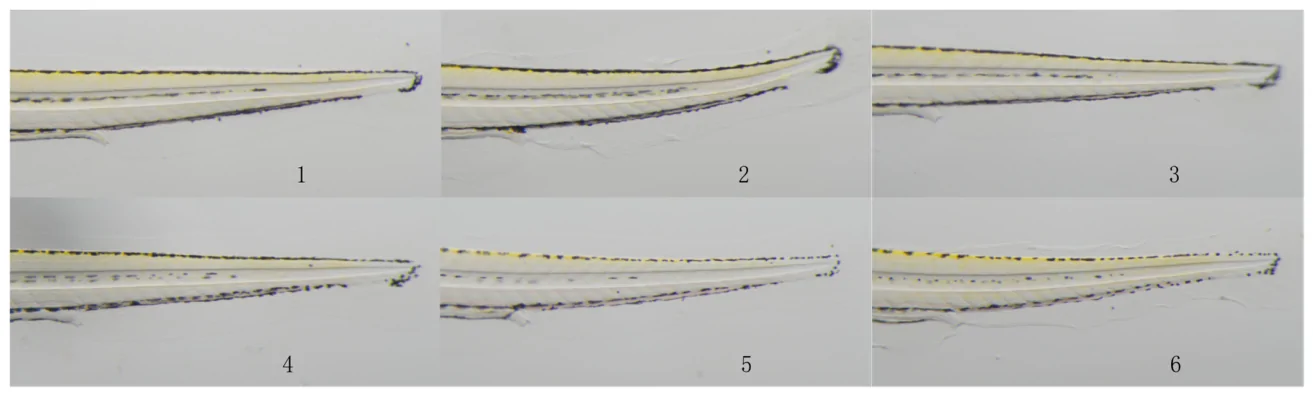
Phenotypes of zebrafish embryos after sample treatment-Tail view. Note: 1—Normal control group; 2—Solvent control group; 3—borneol mandelate 3.29 μM; 4—borneol mandelate 9.87 μM; 5—borneol mandelate 29.6 μM; 6—borneol mandelate 31.2 μM.

**Figure 9:**
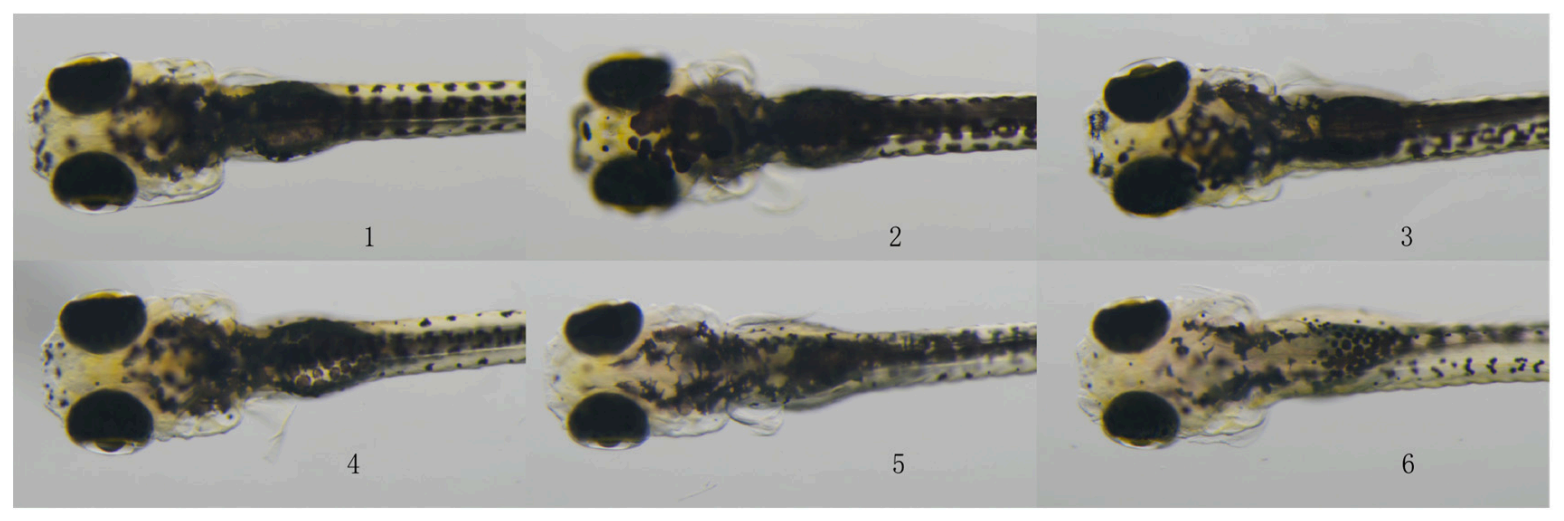
Phenotypes of zebrafish embryos after sample treatment-Dorsal view. Note: 1—Normal control group; 2—Solvent control group; 3—borneol mandelate 3.29 μM; 4—borneol mandelate 9.87 μM; 5—borneol mandelate 29.6 μM; 6—borneol mandelate 31.2 μM.

**Figure 10:**
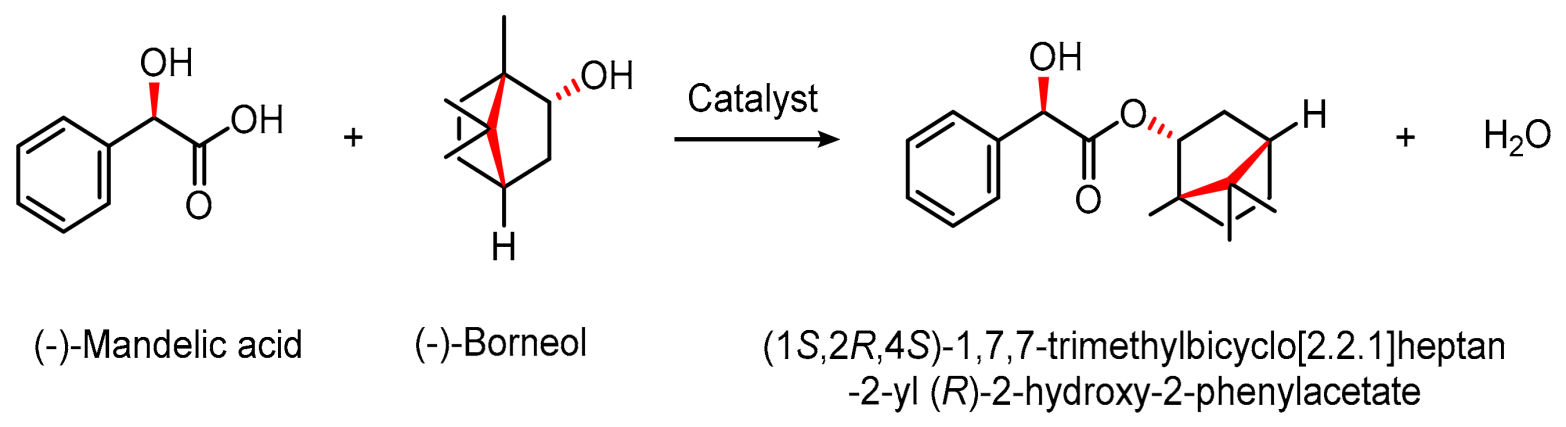
Reaction equation for the synthesis of borneol mandelate.

**Table 1:** Synthesis reactions under different catalyst conditions.

No.	Catalyst	Product Yield (%)	The GC Content of Borneol in the Product (%)	The GC Content of Borneol Mandelate (%)
1	titanium sulfate	71.1	3.6	84.5
2	boric acid	70.4	16.2	73.8
3	boron oxide	70.6	14.1	75.4
4	sulfuric acid	67.5	0.8	60.3

**Table 2:** Synthesis Reactions under different solvent conditions.

No.	Solvent	Product Yield (%)	The GC Content of Borneol in the Product (%)	The GC Content of Borneol Mandelate (%)
1	Toluene	71.1	3.6	84.5
2	chloroform	85.2	-	86.7
3	Cyclohexane	69.3	15.7	72.9

**Table 3:** Results of catalyst reusability tests.

No.	Product Yield (%)	the GC Content of Borneol in the Product (%)	the GC Content of Borneol Mandelate (%)
1	99.1	-	91.7
2	90.4	-	91.5
3	89.6	-	90.8

**Table 4:** Concentration-mortality of zebrafish after sample treatment (*n* = 30).

Group	Concentration (μM)	Number of Dead Fish at 3 dpf	Number of Dead Fish at 4 dpf	Mortality Rate (%)
Normal control group	-	0	0	0
Solvent control group	-	0	0	0
borneol mandelate	3.90	0	0	0
	7.80	0	0	0
	15.6	0	0	0
	31.2	0	3	10
	62.5	30	/	100
	125	30	/	100

Note: ‘/’, not applicable; all larvae died at 3 dpf, and no survival data were recorded at 4 dpf.

**Table 5:** Statistics of acute toxicity incidence induced by sample (*n* = 30).

Type of Malformation		Normal Control Group	Solvent Control Group	Borneol Mandelate (μM)			
				3.29	9.87	29.6	31.2
Heart	Pericardial edema	-	-	-	-	-	-
	Cardiac arrhythmia	-	-	-	-	-	-
Circulatory system	Increased blood flow	-	-	-	-	-	-
	Decreased blood flow	-	-	-	-	10 (3/30)	19 (5/27)
	Circulation loss	-	-	-	-	-	-
Hemorrhage and thrombosis		-	-	-	-	-	-
Brain	Degeneration	-	-	-	-	-	-
Eye	Smaller eye	-	-	-	-	-	11 (3/27)
Liver	Absence	-	-	-	-	-	-
	Reduced liver size	-	-	-	-	33 (10/30)	44 (12/27)
	Hepatic enlargement	-	-	-	-	-	-
	Liver degeneration	-	-	-	-	-	-
	Delayed yolk-sac absorption	-	-	13 (4/30)	33 (10/30)	100 (30/30)	100 (27/27)
Kidney	Edema	-	-	-	-	-	-
Intestine	Abnormality	-	-	-	-	-	-
Trunk/tail/notochord	Curvature	-	-	-	-	-	7 (2/27)
Muscle/somite	Muscle degeneration	-	-	-	-	-	-
Behavior	Abnormality	-	-	-	-	57 (17/30)	7 (21/27)
Mortality		-	-	-	-	-	10 (3/30)

Note: “-“ indicates no obvious abnormality; the numbers in the table represent incidence rates.

## Data Availability

The data presented in this study are available from the corresponding author upon reasonable request.
